# Mediterranean diet and cancer: epidemiological evidence and mechanism of selected aspects

**DOI:** 10.1186/1471-2482-13-S2-S14

**Published:** 2013-10-08

**Authors:** Giuseppe Grosso, Silvio Buscemi, Fabio Galvano, Antonio Mistretta, Stefano Marventano, Vanessa La Vela, Filippo Drago, Santi Gangi, Francesco Basile, Antonio Biondi

**Affiliations:** 1 Department of Drug Sciences, Section of Biochemistry, University of Catania, Catania, Italy; 2 Department of Internal Medicine, University of Palermo, Palermo, Italy; 3 Department "G. F. Ingrassia" Section of Hygiene and Public Health, University of Catania, Catania, Italy; 4 Department of Eating Disorders Unit, Niguarda Hospital, University of Milan, Milan, Italy; 5 Department of Clinical and Molecular Biomedicine, Section of Pharmacology and Biochemistry, University of Catania, Catania, Italy; 6 Department of General Surgery, Section of General Surgery and Oncology, University Medical School of Catania, Italy

## Abstract

**Background:**

Populations living in the area of the Mediterranean Sea suffered by decreased incidence of cancer compared with those living in the regions of northern Europe and US countries, attributed to healthier dietary habits. Nowadays, we are assisting to a moving away from the traditional Mediterranean dietary pattern, but whether this changing is influencing risk of cancers is still unclear. The aim of the study was to review recent evidence on potential relationship between the adherence to the Mediterranean diet and cancer.

**Discussion:**

The most recent pooled analyses of epidemiological studies supported strongly the hypothesis that the Mediterranean diet may play a role in preventing several types of cancers, especially those of digestive tract, whereas contrasting results were reported for hormone-dependent cancers. Specific aspects of the Mediterranean diet such as high fruit and vegetables and low red processed meat intake may explain such protective effects. Moreover, evidence regarding olive oil and whole grains increase the beneficial effects of such dietary pattern against cancer.

**Conclusions:**

Literature evidence actually demonstrates that the increased adherence to the Mediterranean dietary pattern is beneficial to health across populations and may translate a protective effect with certain cancers.

## Background

Nowadays, the advent of improved screening programs and surgical therapeutic approaches have positively affected survival from many cancers [1-3]. On the other hand, differences according non-modifiable and modifiable factors may affect both stage at diagnosis, comorbidity status, and survival [4,5]. Despite the global cancer burden, only few cancers are genetically determined and the most of cancer genesis and promotion mechanisms depends on environmental factors [6-10]. It has been estimated that many of the cancers occurring today are preventable by applying preventive interventions derived by knowledge that we already have [11]. The selective geographical localization of higher incidence rates of many types of cancers in Western countries is attributable to factors related to voluntary behaviors such as nutrition and physical activity habits [12]. Historically, populations living in the area of the Mediterranean Sea suffered by decreased incidence of cancer compared with those living in the regions of northern Europe and US countries [13]. This singular condition has been attributed to the traditional dietary pattern commonly used by populations living in this area, which has been recognized to protect also from cardiovascular diseases [14]. The Mediterranean diet consists of: (i) fruits, cooked vegetables and legumes, wholegrain and unrefined cereals, (ii) olive oil as primary source of dietary lipids, (iii) nuts, (iv) a moderate intake of wine, especially during meals with fish and seafood, dairy products and eggs finally (v) a low consumption of red meat and sweets. Despite a general definition of the Mediterranean diet may be given, there is no univocal pattern, since a variation among countries exist. The studies conducted to estimate the effects on health of the Mediterranean diet aimed to consider the diet preferably as a whole nutrition pattern rather than referring to the single components of the diet, enhancing the role of interactions between the multiple components of the diet. Nowadays, we are assisting to a moving away from the traditional dietary pattern in these regions, generating several variations of the original pattern, or even a "Westernization" of the diet due to the modernization of the urban areas and a globalization of the lifestyle, including diet. Despite a decrease in the adherence to the Mediterranean diet in Mediterranean countries during the last decades has been observed [15], whether this changing in dietary and lifestyle patterns are influencing risk of cancers is still unclear. Studying cancer causes is challenging because increased risk of disease does not only depend on factors related to diet, rather to lifestyle and cultural models of a population. Moreover, due to the long latent period for a malignancy to develop, identify the specific associations between such factors and cancer over a lifetime is difficult. However, accurate literature reviews may help to point-out hypotheses on potential relationship between lifestyle factors such as dietary habits, and cancer. Evidence from epidemiological studies may provide support to public health recommendations and policies.

## Evidence from epidemiological studies

In the past decades, evidence on the health effects of the Mediterranean diet has been reported in many different cohort studies by suggesting its possible protective role against neoplastic diseases. A recent meta-analysis of prospective cohort studies by Sofi et al. [16] concluded that the Mediterranean diet is responsible of a 6% reduction from death and/or the incidence of neoplastic diseases (cancer incidence or mortality RR 0.94; 95% CI: 0.92, 0.96). Updated reports from a large cohort such as the European Prospective Investigation Into Cancer and nutrition including 335,873 individuals have found a lower overall cancer risk among those with greater adherence to Mediterranean diet (HR 0.96, 95% CI 0.95,0.98) for a two-point increment of the Mediterranean diet score [17]. The apparent inverse association was stronger for smoking-related cancers than for cancers not known to be related to tobacco. In all, 4.7% of cancers among men and 2.4% in women would be avoided in this population if study subjects had a greater adherence to Mediterranean dietary pattern. However, no data was given for specific cancer risk.

More recent epidemiological studies focused on association between the Mediterranean diet and incidence of specific malignancies, the most of them regarding colorectal and breast cancers. Regarding colorectal cancer, an a study conducted on 45,275 participants of the Italian section of the EPIC study followed for a mean of 11.28 years, the Italian Mediterranean Index was inversely associated with cancer risk (HR 0.50, 95% CI: 0.35, 0.71 for the highest category compared to the lowest, P-trend: 0.043). Results did not differ by sex. Highest Italian Mediterranean Index score was also significantly associated with reduced risks of any colon cancer (HR 0.54, 95% CI: 0.36, 0.81), distal colon cancer (HR 0.44, 95% CI: 0.26, 0.75) and rectal cancer (HR 0.41, 95% CI: 0.20, 0.81), but not of proximal colon cancer [18]. In the large cohort of 492,382 subjects belonging to the National Institutes of Health-AARP Diet and Health Study that the MD decreased colorectal cancer risk in males (RR 0.72, 95% CI: 0.63, 0.83) was observed, whereas no association in females was reported [19]. Similar results were found in another study conducted on 32,415 subjects included in the contest of the Prostate, Lung, Colorectal, and Ovarian (PLCO) Cancer Screening Trial which assessed that the Mediterranean dietary pattern was associated with reduced risk of colorectal adenoma, especially in men (overall colorectal adenoma OR 0.79, 95% CI: 0.68, 0.92) [20]. On the contrary, a study conducted on a total of 87,256 women and 45,490 men followed for ≤ 26 years found no association with adherence to the Mediterranean Cancer and protection from colorectal cancer [21].

An investigation conducted among the women included in the EPIC cohort showed that adherence to a Mediterranean diet excluding alcohol was related to a modest reduced risk of breast cancer in postmenopausal women, and this association was stronger in receptor-negative tumors [22]. Similar findings have been reported in 33,731 women from the UK Women's Cohort Study with a non-significant inverse association with increasing adherence to the Mediterranean diet pattern in premenopausal but not postmenopausal women [23] whereas a marginally significant inverse association (HR 0.78 for every 2 points, 95% CI: 0.62, 0.98) among postmenopausal women in the Greek arm of the EPIC study was found [24]. Moreover, although the Minnesota Breast Cancer Family Study did not find an association between MD and breast density, a stratified analysis identified a protective role of the Mediterranean diet among current smokers (beta = -1.68, P = 0.002) but not among nonsmokers (beta = -0.08, P = 0.72; P for interaction = 0.008) [25]. The study of the histological features of the breast cancer observed leaded to contrasting results. Fung et al. observed no association between the diet quality indices and total or positive estrogen receptor (ER+) breast cancer risk with postmenopausal women who scored high in MD adherence score had a lower risk of negative estrogen receptor (ER-) breast cancer (OR 0.79 95% CI: 0.60-1.03) [26]. Contrarily, a French study reported an increase of breast cancer risk with the Western diet for ER+/positive progesterone receptor (PR+) tumors (HR 1.20, 95% CI: 1.03-1.38), and a negative association of the MD for breast cancer risk (HR 0.85, 95% CI: 0.75-0.95), especially for ER+/PR- tumors [27].

Among the other cancer incidences explored within large epidemiological prospective cohort studies, some investigation regarded prostate, gastric, and endometrial cancers. A study aimed to explore the association between adherence to a relative Mediterranean diet and incident gastric adenocarcinoma within the EPIC cohort reported that a 1-unit increase in the Mediterranean diet score was associated with a decreased risk of cancer of 5% (95% CI: 0.91, 0.99) [28]. No significant association was also observed between a Mediterranean diet and total prostate cancer or between any of the indices and advanced or fatal prostate cancer in a cohort of 293,464 US men in the National Institutes of Health (NIH)-AARP Diet and Health Study [29]. In individual component analyses, the fish and ω-3 fatty acids were inversely associated with fatal prostate cancer (HR = 0.79, 95% CI: 0.65, 0.96, and HR = 0.94, 95% CI: 0.90, 0.98, respectively). Regarding the incidence of endometrial cancer, a study conducted in San Francisco on woman aged 35-79 showed that a greater consumption of the Western diet was associated with a 60% increase in the risk cancer but no association with endometrial cancer was found with the Mediterranean diet [30].

The association between the Mediterranean diet and cancer has been studied in a series of case-control studies conducted on breast, colon, and upper aero-digestive tract cancers [31-33]. The one of Demetriou et al. [31] did not report any association between breast cancer and Mediterranean diet, although higher consumptions of vegetables, fish and olive oil, were independently associated with decreased risk of disease. Contrarily, a study including two case-control groups of Hispanic (757 cases, 867 controls) and non-Hispanic white women (1524 cases, 1598 controls) from the Four-Corners Breast Cancer Study reported that the Mediterranean dietary patterns were associated with lower risk of breast cancer (OR 0.76, 95% CI: 0.63, 0.92) [34]. Another series of studies focused on the mediating effect of Mediterranean diet on a variety of lifestyle behaviors and colorectal cancer, have found that increase in the adherence to the Mediterranean diet was associated with lower likelihood of colorectal cancer whereas a positive association with alcohol intake and smoking habit was reported [32,33]. Finally, the study conducted in the context of the European alcohol-related cancers and genetic susceptibility in Europe project, reported that stricter adherence to the Mediterranean diet was associated with a substantial and significant decrease in UADT cancer risk (30 % for a two-unit increase in score) [35], confirming results of a work reporting data from three case-control studies conducted in Italy between 1992 and 2000 in which an OR of 0.40 for oral and pharyngeal, 0.26 for esophageal, and 0.23 for laryngeal cancer in subjects more adherent to the Mediterranean dietary pattern was found [36].

## Selected components of Mediterranean diet protecting against cancer

### Fruit and vegetables

The role of the main components of the Mediterranean diet in several common epithelial cancers, including digestive and selected non-digestive tract neoplasms, have been showed in a report of La Vecchia [37] reviewing a series of case-control studies conducted in Italy between 1983 and 1998 over 12,000 cases of 20 cancer sites and 10,000 controls. The relative risk (RR) for most epithelial, digestive tract, breast, female genital tract, urinary tract cancers was decreased with increasing vegetable and fruit consumption, probably due to the beneficial effects of a number of antioxidants and other micronutrients which showed themself an inverse relationship with cancer risk [38].

Raw and fresh vegetables, leafy green vegetables, cruciferous vegetables, carrots, broccoli, cabbage, lettuce, and raw and fresh fruit (including citrus fruit and tomatoes) appear to be protective against cancer due to their high content of antioxidants compounds, such as carotenoids, vitamin C, vitamin E, selenium, dietary fiber (and its components), dithiolthiones, glucosinolates (isothiocyanates and indoles), polyphenols, protease inhibitors, allium compounds, plant sterols, and limonene [39,40]. A high intake of glucosinolate-containing cruciferous vegetables, such as Brussels sprouts (*Brassica oleraceae*), has been linked to a decreased cancer risk [41]. Indoles and isothiocyanates, two major groups of glucosinolate breakdown products, attenuate the effects of polycyclic aromatic hydrocarbons (PAHs) and nitrosamines under experimental conditions [42]. In addition, indole-3-carbinol (I3C), a naturally occurring component of *Brassica vegetables*, such as broccoli, cabbage, and Brussels sprouts, has been found to prevent the progression of different cancers and in particularly induces a G(1) cell-cycle arrest of human breast cancer cells. Moreover, indole-3-carbinol (I3C) has significant therapeutic potential for treatment of human cancers associated with high levels of elastase [43]. This anti-tumoral effect is expressed by a noncompetitive inhibition of elastase enzymatic activity. Indeed, high accumulation of cyclin E protein is associated with cancer cell proliferation, poor clinical outcomes, decreased response to chemotherapy, reduced response to endocrine treatment and a reduced survival rate in some human cancers [44].

Citrus fruit, especially blond and red oranges (*Citrus sinensis*), contains a high amount of antioxidant components including polyphenols, flavanones, anthocyanins, hydroxycinnamic acids, and ascorbic acid (depending on the variety of fruit) [45]. Laboratory studies have showed that citrus flavonoids and anthocyanins are able to inhibit the growth of several tumors, including breast and colorectal cancer [46,47] through inhibiting multiple cancer-related biological pathways, such as carcinogen bio-activation, cell-signaling, cell cycle regulation, angiogenesis, and inflammation [48,49]. Citrus flavonoids have been hypothesized to inhibit the matrix metalloproteinase (MMP) secretion, migration, invasion and adhesion [50-52], and to have pro-apoptotic activity [53-56].

Tomatoes are undergoing particular attention because they clearly characterize the Mediterranean diet. Eating tomatoes has been associated with reduced risks of some types of cancer and other diseases [57]. Their constituents such as carotenoids and flavonoids contribute to the prevention of ultra violet ray damage in humans [58] and are efficient antioxidants capable of scavenging reactive oxygen species generated under conditions of photo-oxidative stress [59]. Lycopene, red pigment present in many red fruits and vegetables, is the major carotenoid of the tomato and is a very efficient singlet oxygen quencher in the group of carotenoids [58]. Tomatoes and related tomato products are the major source of lycopene compounds, and are also considered an important source of carotenoids in the human diet. Industrial and thermal processing (bleaching, retorting, and freezing processes) generally causes some loss of lycopene in tomato-based foods, although lycopene bioavailability in processed tomato products is higher than in unprocessed fresh tomatoes [60]. Food processing may improve lycopene bioavailability by breaking down cell walls, which weakens the bonding forces between lycopene and tissue matrix, thus making lycopene more accessible [61]. Supplementation of tomato products, containing lycopene, has been assessed to have similar effects on health than consuming tomato. Indeed, such products supplementation lower biomarkers of oxidative stress and carcinogenesis [62] and protect the skin against UVR-induced effects (i.e., erythema) [63,64]. Moreover, tomato extract has been shown to ameliorate tissue damage, to decrease the risk of many chronic diseases including several types of cancer (i.e., prostate cancer) [65,66]. Purple tomatoes, highly enriched with anthocyanins, have been shown to prolong life under experimental conditions suggesting that they have additional health-promoting effects [67].

### Fish vs. meat

Frequent fish intake has been related to decreased risk of several neoplasms whereas red meat intake demonstrated detrimental effects, especially increasing the risk of colorectal cancer [68], through production of N-nitroso compounds and, when cooked at high temperatures, heterocyclic amines and polycyclic aromatic hydrocarbons (which are known carcinogens) [69]. Red meat and processed meat intake has been hypothesized to increase breast cancer risk but while both case-control and ecologic studies have supported a positive association, prospective cohort studies have been inconsistent [70]. Further investigations of potential effect modifiers, such as analyses by hormone receptor or menopausal status, may provide valuable insight to potential patterns of associations. Indeed, a meta-analysis including only studies conducted on premenopausal women demonstrated a pooled RR of 1.24 (95% CI 1.08, 1.42) [71]. Among the more evident associations, meat and processed meat intake has been related with esophageal [72,73], lung [74], pancreatic [75], bladder [76] cancers, whereas inconclusive results were reported for prostate [77], kidney [78], and ovarian [79] cancers.

### Whole-grain

Consumption of refined and whole grains, in form of bread, pasta, and rice, is a major characteristic of MD and has been associated with reduced cancer risk, including those of the upper aero-digestive tract, stomach, colorectum, liver, breast, ovary, bladder and kidney [80], while refined grain intake was associated with an increased risk of stomach, colorectal, upper aero-digestive tract and thyroid cancers [81]. The beneficial role of whole-grain has been hypothesized to be due to their high content of fibres, which OR of colon and rectal cancers were below unity for high intakes of most types of dietary fibres, especially those contained in rice [82], whereas no appreciable differences emerged between the two sites [83-85]. Nevertheless, high intakes of most types of dietary fibres have been associated with lower odds also for upper aero-digestive tract neoplasms [86,87]. Dietary fibers may act on cancer risk by improving faecal bulking and satiety, viscosity and short chain fatty acid production, enhancing fermentation of metabolites. Fibres may protect from colorectal cancer by increasing stool bulk, thus reducing transit time and contact of carcinogens with the colonic mucosa or by acting as probiotic, namely a nondigestible food ingredient whose beneficial effects on the host result from the selective stimulation of growth and/or activity of members of the bacterial community that inhabits the human bowel [88]. The fermentation of dietary fibre by the microflora enhances the levels of effective metabolites which are potentially protective against colon cancer inhibiting cell growth and promoting apoptosis as well as differentiation [89,90].

Another hypothesis investigated on the possible mechanism of cancer promotion related to refined and whole grain foods, was their connection with the dietary glycaemic index (GI) (an indicator of the rate of adsorption of carbohydrates and, hence, a measure of insulin demand) and glycaemic load (GL) (which combines the quality as well as the quantity of carbohydrate consumed). Both GI, and in particular GL, have been suggested to be relevant factors in the cancer incidence due to the potential role of insulin and insulin grow factor (IGF) in cancer promotion [91,92]. With reference to digestive tract cancers, there was a direct association of colon and gastric cancer with such indices; among female hormone-related neoplasms, direct associations with breast, ovarian and endometrial cancers; among upper aero-digestive tract neoplasms, an association was found for those of the oral cavity/pharynx, esophagus and larynx; finally, a significant association was found for thyroid and prostate cancers [84]. Results of recent meta-analyses of prospective cohort studies reported a pooled RR for all diabetes-related cancers of 1.07 (95% CI: 1.04, 1.11) for GI and 1.02 (95% CI: 0.96, 1.08), whereas the analysis of site-specific cancer risks revealed a significant associations for GI in relation to breast cancer (RR 1.06, 95% CI: 1.02, 1.11) and colorectal cancer (RR 1.08, 95% CI:1.00, 1.17) and for GL to endometrial cancer (RR 1.21, 95% CI: 1.07, 1.37) [93] whereas no significant risk of digestive tract neoplasms was found [94]. The role of fibre in preventing chronic disease also depends on their numerous bioactive compounds with antioxidant and anti-carcinogenic properties, especially those in the bran and germ (minerals, trace elements, vitamins, carotenoids, polyphenols and alkylresorcinols), the properties of which have been described above. Whole-grain wheat is also a rich source of methyl donors and lipotropes (methionine, betaine, choline, inositol and folates) that may be involved in cardiovascular and hepatic protection, lipid metabolism and DNA methylation [95].

### Olive oil

The association of olive oil and other mono- and unsaturated fats with many types of cancer has been reviewed, reporting a favorable effect on breast, ovarian, colorectal, but mostly of upper aero-digestive tract cancers [84]. A recent meta-analysis of 13800 patients and 23340 controls in 19 observational studies concluded that higher intake of olive oil intake was associated with lower odds of having any type of cancer (log OR -0.41, 95% CI: -0.53, -0.29), breast cancer (log OR -0.45, 95%CI: -0.78, -0.12), and a cancer of the digestive system (log OR -0.36, 95% CI: -0.50, -0.21), compared with the lowest intake [96]. Experimental and human cellular studies have provided new evidence on the potential protective effect of olive oil on certain cancers such as breast, colorectal and prostate cancers [97]. Olive oil has been suggested to inhibit colon cancer development by inducing apoptosis in large intestinal cancer cells and down-regulating the expression of cyclooxygenase 2 (COX-2) and Bcl-2 proteins that have a crucial role in colorectal carcinogenesis [98]. Among its potentially health-promoting components, tyrosol and hydroxytyrosol have been demonstrated to decrease glutathione (GSH), the activation of the transcription factor Nuclear Factor-κB and cell death which may be implicated in the carcinogenetic processes [99].

### Red wine

Wine, especially red wine, is rich in several plant compounds with a wide range of health properties. Whether moderate intake of red wine may protect against cancer is still controversial [100]. Many of the beneficial properties of red wine depend on its content of resveratrol (3,4',5-trihydroxy-trans-stilbene), a phytoalexin found also in grape skins, peanuts and berries, that may act as an antioxidant and cancer chemopreventive agent thanks to its ability of inhibiting tumor initiation, promotion, and progression [101]. Indeed, several studies have shown that resveratrol and some of its analogs interfere with signal transduction pathways, modulate cell cycle-regulating proteins, and are a potent inducer of apoptosis in multiple carcinoma cell lines [102]. Recently, the chemopreventive properties of resveratrol have been associated with the inhibition of Nuclear Factor-kB activity [103]. In addition, resveratrol exhibits anti-inflammatory, growth-inhibiting activity and immunomodulation properties [104]. Interestingly, the aforementioned health benefits of resveratrol against cancer produced in the laboratory setting cannot be translated in effects on free-living population. Indeed, some cohort studies conducted on lung [105], colorectal [106], and prostate [107] cancers, reported that red wine does not contribute appreciably to the etiology or protection of such malignancies. It may be speculated that the beneficial effects of antioxidant compounds contained in red wine may be offset by the detrimental effects of alcohol.

## Conclusions

Several aspects of the MD may lead to some health benefits that reflect a higher survival and a lower incidence of cancer. However, limitations of literature studies have to be considered. Firstly, studies exploring the adherence to the MD and cancer incidence or mortality, have a methodological limitation due to the longer follow-up period, during which the latency of dietary influences on the risk of death may be influenced by changes in dietary habits, if diet is assessed at enrolment and if a cumulative exposure to a more or less healthy diet is considered. Moreover, some further confounding factors such as, the effect of dietary supplements, may lead to sure bias if studies do not control or results are not adjusted for these variables. Finally, possible measurement error and recall bias should be taken in to account. Another limitation of some studies is the availability of information on non-dietary variables such as socioeconomic and cultural status. In fact, the only variable taken in account was the latest educational achievement which is both objectively ascertainable and internationally applicable, but it is not comprehensive of other factors influencing the lifestyle. Another non-dietary factor that should be considered in these kinds of studies and is often missing or not homogeneously measured is the physical activity level. In fact physical activity is an independent risk factor for many types of cancer. Despite these limitations, literature evidence actually demonstrates that the increased adherence to the Mediterranean dietary pattern is beneficial to health across populations and may translate a protective effect with certain cancers.

## Competing interests

The authors declare that they have no competing interests.

## Authors' contributions

GG: conception and design, drafting the manuscript; AM, SM, VLV: drafting the manuscript; SB, FG, FD, FB, SG, AB: critical revision, given final approval of the version to be published.

## Declarations

Funding for this supplement has come from University funds.

This article has been published as part of *BMC Surgery *Volume 13 Supplement 2, 2013: Proceedings from the 26th National Congress of the Italian Society of Geriatric Surgery. The full contents of the supplement are available online at http://www.biomedcentral.com/bmcsurg/supplements/13/S2
